# Supplementary material for: Netcombin: An algorithm for constructing optimal phylogenetic network from rooted triplets
Source: PLoS One. 2020 Sep 18;15(9):e0227842. doi: 10.1371/journal.pone.0227842 (PMC7500971; doi:10.1371/journal.pone.0227842)
Supplement: S4 File — (ZIP) [file pone.0227842.s004.zip › SequenceData-Triplet/sequenceData-10-20-40-Triplet2/data-(sample 10- length 500- num seq 40- m rate 10^-6)/2 seq.pdf]

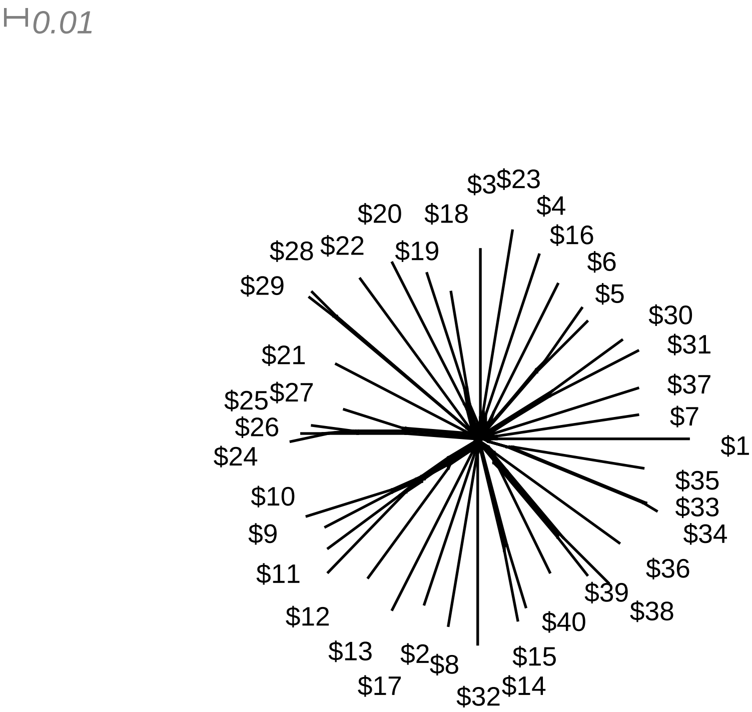
0.01
$3
$23
$4
$20
$18
$16
$28
$22
$19
$6
$29
$5
$30
$31
$21
$37
$25
$27
$7
$26
$1
$24
$35
$10
$33
$34
$9
$36
$11
$39
$38
$12
$40
$13
$2
$8
$15
$17
$14
$32
